# Maximizing Electrokinetic Energy Conversion via the Intersecting Asymptotes Method

**DOI:** 10.1038/s41598-018-37360-6

**Published:** 2019-01-24

**Authors:** Abraham Mansouri, Larry Kostiuk

**Affiliations:** 1 Department of Mechanical Engineering, Higher College of Technology, Dubai, 15825 UAE; 2 Department of Mechanical Engineering, University of Alberta, Edmonton, Alberta T2G 2G8 Canada

## Abstract

It has been shown in earlier studies that the maximum electrokinetic conversion efficiency between flow and electric work (e.g., electrokinetic power generation) occurs when electric double-layer (λ) overlaps and there is no electroneutral zone in a nanometer-scale channel. This result has been shown through cumbersome and lengthy numerical and theoretical studies. The case is made here that complications associated with solving the coupled set of governing equations i.e. Poisson, Nernst-Planck, and Navier-Stokes (PNPNS) could be drastically reduced to a two-step solution by method of intersecting asymptotes.

## Introduction

The theory of electrokinetic transport in micro/nano channels, particularly in the analysis related to the calculation of maximum electrokinetic energy conversion has been developed over the years, with several key studies^1–12^. The pressure-driven electrokinetic flows in such geometries can be modeled in the framework of a coupled problem between modified Navier-Stokes and Poisson-Nernst-Planck equations, where the electrochemical transport of electrolyte solution and the ion transports by convection, diffusion, and migration are described, respectively. In this paper we employ the method of intersecting asymptotes to simplify the electrokinetic energy conversion problem. This method has been employed to explore complicated multi-physics phenomena and it is helpful to describe the phenomenon in the simpler extremes (asymptotes) in which it may manifest itself. The intersection of asymptotes method provides a direct and shortcut to capture the most important characteristics of the problem^13^. The method consists of only two steps, initially to find the functional form of the solution in the two possible extremes, in case of the current problem *i*.*e*. electrokinetic transport in a nanochannel with radius of “r” the two extreme cases are: thin (λ ≪ r) and thick (λ ≫ r) double layer assumptions. In the second step, the two asymptotes graphically are intersected to determine the radius of a nanochannel that identifies a potential extremum (in this case a maximum) in the electrokinetic energy conversion efficiency.

In electrokinetic power generation the source of current is the transport of ions by pressure driven flows. The origin of such streaming current is the electrical state of the fluid-substrate interface that creates a spatial distribution in the free charge density, which is then transported by the fluid velocity, *u*, parallel to the walls. The streaming current is the product of velocity field and net charge density and is given by.

1 $${I}_{{\rm{streaming}}}=2\pi {\int }_{0}^{r}\,(r)\cdot {\rm{u}}(r)\cdot r\cdot dr$$

The description of the free charge density *ρ*(*r*) depends upon whether we apply the thin or thick double-layer assumptions. We begin by invoking the thin double-layer assumption and, for simplicity, the Debye-Huckel approximation in which the electrical potential within the double layer is assumed to be small (<25.7 mV at 25C for water-glass interface). The streaming current can then be written as a function of either constant surface change density or constant surface potential as below^14,15^.

2 $${I}_{\mathrm{streaming} \mbox{-} \mathrm{thin}}=\frac{\pi {r}^{2}\Delta P\varepsilon \zeta }{\mu L}=\frac{\pi {r}^{2}\Delta p{\rm{\lambda }}\sigma }{\mu L}\sim {r}^{2}{\rm{\lambda }}\sigma ;\,{\rm{\lambda }}\ll {\rm{r}}$$

where *ε* is the fluid permittivity, ζ is the zeta potential, *σ* is the surface charge density, ${\rm{\lambda }}\sim \sqrt{\frac{\varepsilon {K}_{b}T}{2{e}^{2}{n}_{\infty }}}$ is the Debye length and *ε*ζ = *σ*λ, µ is the viscosity, *ΔP*/*L* is pressure gradient, T is the temperature, *K*_*b*_ is Boltzmann constant and r is the radius of the channel. We now invoke the other extreme *i*.*e*. the thick double-layer assumption where the total surface charge on the nanochannel should be balanced by the excess of counterions within the electrolyte solution and streaming current can be then written as^16^.

3 $${I}_{\mathrm{streaming} \mbox{-} \mathrm{thick}}=\frac{\pi {r}^{3}\Delta P\sigma }{4\,\mu L}\sim {r}^{3}\sigma ;\,{\rm{\lambda }}\gg r.$$

The theoretical electrokinetic energy conversion efficiency of fluid flow to electric power in a single nanochannel can be obtained by dividing the rate of electric work over the rate of flow work. The rate of electric work is expressed by $\,{\dot{w}}_{el}={I}^{2}R$ where I is the streaming current and R is the total electrical resistance of the nanochannel. The electrical resistance of a nanochannel is a function of electrolyte conductivity (*K*) and geometry and its expressed as $R=\frac{L}{\pi {r}^{2}K}$ where L is the length of nanochannel. The rate of flow work is the product of volumetric flow rate (Q) and pressure difference across the nanochannel ${\dot{w}}_{fl}=\Delta Q\Delta P$. The theoretical electrokinetic energy conversion is simply expressed as $\eta =\frac{{\dot{w}}_{el}}{{\dot{w}}_{fl}}$. The quest for optimal/maximizing energy conversion efficiency has been long and drives us to seek simpler solutions in this study. First consider the thin-double layer assumption where double layer thickness is much smaller than the radius of the channel. It can be shown that for a thin double layer, the electric conductivity of the electrolyte solution is inversely proportional to the square of double layer thickness $K\sim \frac{2D{e}^{2}{n}_{\infty }}{{K}_{b}T}\sim \,\frac{\varepsilon D}{{{\rm{\lambda }}}^{2}}\sim \frac{1}{{{\rm{\lambda }}}^{2}}$ where D is the ion diffusivity^17,18^. The volumetric flow rate is estimated by Hagen-Poiseuille equation $Q=\,\frac{{{\rm{\pi }}r}^{4}{\rm{\Delta }}{\rm{P}}}{8\,\mu {\rm{l}}}\sim {r}^{4}$ as velocity is obtained by Poiseuille equation $V\sim \frac{1}{4\,\mu }({r}^{2}-{a}^{2})\frac{\Delta p}{L}\sim {r}^{2}$ and surface area *A* ~ *r*^2^ in a circular channel. Substituting above correlations into the efficiency equation, it can be further simplified as below.

4 $$\eta =\frac{{I}^{2}R}{\Delta P\Delta Q}=\frac{{(\frac{\pi {r}^{2}\Delta P{\rm{\lambda }}\sigma }{\mu L})}^{2}(\frac{L}{\pi {r}^{2}K})}{\Delta PV\pi {r}^{2}}\sim \frac{({r}^{4}{{\rm{\lambda }}}^{2}{\sigma }^{2})(\frac{{{\rm{\lambda }}}^{2}}{{r}^{2}})}{{r}^{4}}\sim \frac{{{\rm{\lambda }}}^{4}{\sigma }^{2}}{{r}^{2}}\sim \frac{{{\rm{\lambda }}}^{2}{(\varepsilon \zeta )}^{2}}{{r}^{2}}\sim f(\frac{{\rm{\lambda }}}{r}){}^{2};\,{\rm{\lambda }}\ll r.$$

The efficiency for thin-double layer assumption (*i*.*e*. a microchannel) can be expressed as a function of ${(\frac{{\rm{\lambda }}}{r})}^{2}$, it increases rapidly with Debye screening length and decrease with channel radius and the maximum power varies as *P*_*max*_ ~ *r*^2^. Now considering the thick-double layer assumption where double layer thickness is much larger than the radius of channel. It can be shown that for a thick-double layer, due to balance between surface charges and counterions inside the nanochannel the electric conductivity of the electrolyte solution is directly proportional to the surface charge on the channel wall and is inversely proportional to channel radius $K\sim \frac{\sigma }{r}$ ^19^. Substituting previous correlations into the efficiency equation for thick double layer assumption, it can be further simplified as below.

5 $$\begin{array}{rclcl}\eta  & = & \frac{{I}^{2}R}{\Delta P\Delta Q}=\frac{{(\frac{\pi {r}^{3}\Delta P\sigma }{4\mu L})}^{2}(\frac{L}{\pi {r}^{2}K})}{\Delta PV\pi {r}^{2}}\sim \frac{{({r}^{3}\sigma )}^{2}(\frac{r}{{r}^{2}\sigma })}{{r}^{4}}\sim \frac{({r}^{6}{\sigma }^{2})(\frac{1}{r\sigma })}{{r}^{4}} & \sim  & r\sigma \sim f\,{(\frac{{\rm{\lambda }}}{r})}^{-1};\,{\rm{\lambda }}\gg r.\end{array}$$

The efficiency for the thick-double layer assumption (*i*.*e*. a nanochannel) can be expressed as a function of ${(\frac{{\rm{\lambda }}}{r})}^{-1}$, and contrary to thin-double layer assumption, it decreases with Debye length and increases with the nanochannel radius and the maximum power varies as *P*_*max*_ ~ *r*^5^. Similar to a circular nanochannel, the efficiency of electrokinetic energy conversion in a nanoslit is directly proportional to both nanoslit height and surface charge density^2^.

**Figure 1:**
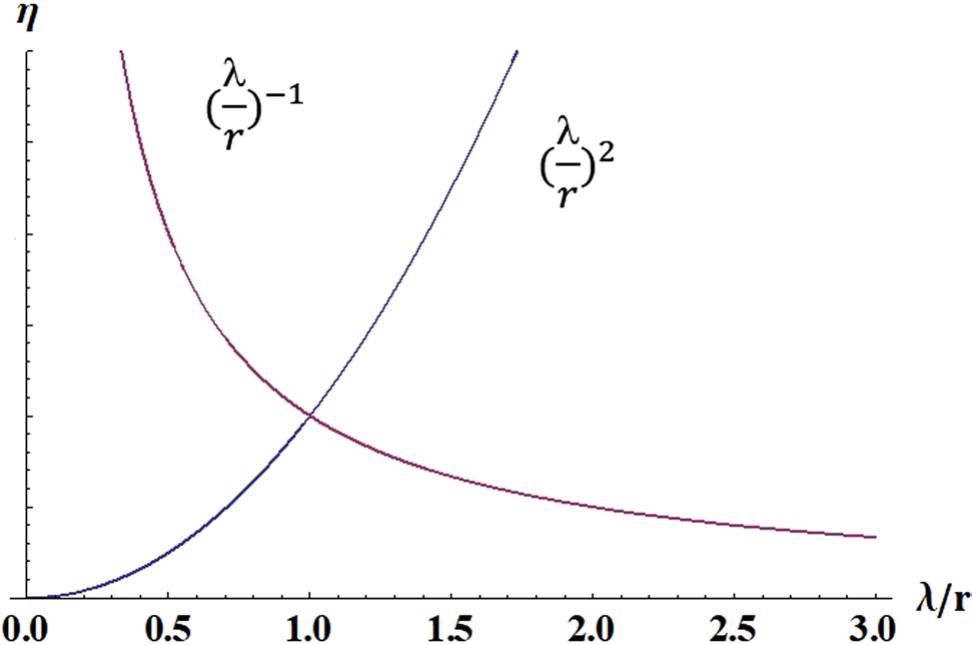
Intersection of asymptotes method, the maximum of electrokinetic energy conversion efficiency occurs where electric double layer overlaps.

What we have determined so far are the two asymptotes of the curves of electrokinetic energy conversion efficiency (η) versus non-dimensional Debye length $(\frac{{\rm{\lambda }}}{r})$ with respect to channel radius. As shown in Fig. 1 by plotting the two asymptotes we find the maximum electrokinetic energy conversion efficiency occurs within the range of double-layer overlap i.e. *r*_*optimum*_ ~ λ. The extended electric double layer enhances the concentration of counter-ions in the center of the nanochannel where the velocity fields are extreme thus maximizing the efficiency^1–12^. Daiguji *et al*. through a detailed numerical study showed the efficiency of electrokinetic energy conversion in a nanoslit is also maximized when Debye length is in the order of half of channel height. In double layer overlap regime, a unipolar electrolyte solution is generated inside the nanochannel to sustain the electrical neutrality^2^. In Fig. 1 the thin and thick models become increasingly inaccurate as they approach the other model’s point of validity, yet they produce the same result at value of unity on x-axis. Similar analysis can be performed for electroosmotic pumping (reverse effect of streaming potential/power generation, *i*.*e*. generating fluid flows by applying potential differences) where $\,{\eta }_{pumping}=\frac{\Delta P\Delta Q}{{I}^{2}R}$. Yao *et al*. showed that maximum electrokinetic energy conversion efficiency in electroosmotic pumping also occurs in double-layer overlap regimes^20^. In addition, non-equilibrium thermodynamic theory has shown that the maximum efficiency in either direction of electrokinetic energy conversion is the same^21^.

We have shown through a simple but effective mathematical method (intersecting asymptotes) that the electrokinetic energy conversion efficiency can be maximized in the range of double layer overlap. While our findings are in excellent agreement with previous detailed modeling studies, we showed that the method of intersecting asymptotes is a powerful and useful tool for simplifying sophisticated multi-physics systems. We anticipate that further applications of this method will facilitate the solution of electrokinetic optimization problems.
