# Publisher Correction: Maximizing Electrokinetic Energy Conversion via the Intersecting Asymptotes Method

**DOI:** 10.1038/s41598-019-41669-1

**Published:** 2019-04-11

**Authors:** Abraham Mansouri, Larry Kostiuk

**Affiliations:** 1 Department of Mechanical Engineering, Higher College of Technology, Dubai, 15825 UAE; 2 Department of Mechanical Engineering, University of Alberta, Edmonton, Alberta T2G 2G8 Canada

Correction to: *Scientific Reports* 10.1038/s41598-018-37360-6, published online 24 January 2019

This Article contains an error in Equation 1.

$${I}_{{\rm{streaming}}}=2\pi {\int }_{0}^{r}\,(r)\cdot {\rm{u}}(r)\cdot r\cdot dr$$

should read:

$${I}_{{\rm{streaming}}}=2\pi {\int }_{0}^{r}\,\rho (r)\cdot {\rm{u}}(r)\cdot r\cdot dr$$

